# The Quality of Providing Dental Services in the School of Dentistry and Private Clinics in Shiraz, Based on the SERVQUAL Model 

**DOI:** 10.30476/dentjods.2024.99413.2149

**Published:** 2025-06-01

**Authors:** Maryam Bakhtiar, Mohsen Sharif Zadeh Ardakani, Mohammad Reza Mokarram, Samaneh Ansarifar, Mehrdad Vossoughi

**Affiliations:** 1 Dept. Oral and Dental Disease Research Center, Dept. of Dental Public Health, School of Dentistry, Shiraz University of Medical Sciences, Shiraz, Iran.; 2 PhD Student in Dental Public Health, Dept. of Dental Public Health, School of Dentistry, Shiraz University of Medical Sciences, Shiraz, Iran.; 3 Undergraduate Student, Student of Committee Research, School of Dentistry, Shiraz University of Medical Sciences, Shiraz, Iran.

**Keywords:** Dental School, Private clinic, SERVQUAL model

## Abstract

**Background:**

The quality of dental services is an important factor in patient satisfaction and health outcomes. However, there is a lack of studies, which compare the quality of dental services provided in different settings such as dental schools and private clinics and measure the gap between patients' expectations and perceptions.

**Purpose:**

This study aims to assess and compare the quality of dental services in Shiraz Dental School and private clinics using the SERVQUAL model and identify the dimensions that affect patient satisfaction.

**Materials and Method:**

In this cross-sectional descriptive study, 440 patients of the Shiraz dental school and private clinics in Shiraz participated in answering the SERVQUAL questionnaire to measure the perceptions and expectations of patients in six dimensions (tangibility, reliability, responsiveness, assurance, empathy, and access).

**Results:**

The mean gap score was (-1.27±0.59) for private clinics and (-0.40±0.45) for dental school, which was significant (*p*< 0.001) between expectations and perceptions in all dimensions in both settings. In private clinics, the largest gap was in the empathy dimension (-1.64±0.65) and the smallest gap was in the assurance dimension (-1.05±0.64). In the Shiraz dental school, the largest gap was in the tangibility dimension (-0.72±0.60) and the smallest gap was in the assurance dimension (-0.59±0.65).

**Conclusion:**

The patients were satisfied with the services provided in the Shiraz dental school and private clinics of Shiraz, but the expectations of the patients were not met in all dimensions, which require periodic evaluations along with policies to respond to the needs and expectations of the patients in providing services in both sectors.

## Introduction

In today's competitive world, large companies and organizations are constantly striving to improve and evaluate their strategies and policies. This applies to health systems as well, as the World Health Organization emphasized the importance of enhancing the performance of health systems in its 2000 report. A key factor in evaluating and improving the quality of an organization is the customer's perspective on service quality (SERVQUAL) [ 1 ]. Therefore, patient satisfaction with the health and treatment services provided by medical centers and institutions is an essential tool for assessing the quality of care [ 2 - 4 ], which is one of the main components of healthcare quality along with treatment success [ 4 - 5 ]. Measuring the quality of medical services based on patients' opinions and feedback has become increasingly popular in recent years [ 4 ]. However, quality is a complex and multidimensional concept that is hard to define objectively. According to the American society for quality (ASQ), quality is the ability to produce a product or provide a service that meets the needs and expectations of customers [ 6 ]. In the context of health care services, patient satisfaction is considered a suitable indicator of the quality of treatment and the doctor-patient relationship, which should be periodically monitored and evaluated in medical centers to identify and eliminate possible shortcomings and problems [ 7 ]. A useful tool for measuring patients' expectations and perceptions of the SERVQUAL model, which can help managers of healthcare systems recognize their strengths and weaknesses. By addressing SERVQUAL gaps, managers can enhance the perceived SERVQUAL and consequently increase customer satisfaction [ 1 , 5 ]. The SERVQUAL model was developed in the 1980s as a result of scientific studies in the field of SERVQUAL, and it consists of five dimensions: tangibility, reliability, responsiveness, assurance, and empathy [ 1 , 8 ]. Since 1991, SERVQUAL has been widely used as a measurement tool, especially in the field of medical services [ 1 , 9 - 11 ]. In the field of dental services, Davies and Ware developed a tool to measure patients' satisfaction with dentists' services based on five dimensions: access, facilities, cost, care and quality [ 7 ]. Ghanbarzadegan *et al*. [ 1 ] in 2016 conducted a study on 180 patients from different clinical departments of Rafsanjan dental school and reported a gap of -1.64 ± 0.65 between expectations and perceptions in all dimensions of the questionnaire. Rocha *et al*. [ 12 ] found that women were more dissatisfied with the tangibility and reliability dimensions, elderly people with the empathy dimension, and people with low parental literacy with the assurance and empathy dimensions. This study aims to assess and compare the quality of dental services provided in Shiraz dental school and private clinics using the SERVQUAL model and to identify the dimensions that affect patient satisfaction. 

## Materials and Method

###  Study design and population

This study was a cross-sectional descriptive study that aimed to assess and compare the quality of dental services provided in Shiraz dental school and private clinics using the SERVQUAL model. The study population consisted of adult patients (aged 18-65) who visited the Shiraz dental school and private clinics in Shiraz city in 2018. The sample size was calculated at 240 patients for dental school and 200 patients for private clinics (a total of 440 patients). The study included the patients who visited the dental school in the second half of the academic year 2017-2018, were between 18 and 65 years old, agreed to participate in the study, and were able to answer the questions without any difficulty. The study excluded patients who were treated in the pediatric, orthodontic, and pathology departments, because they were either too young or had long treatment duration, or did not have direct contact with the service providers. The study also excluded patients who were younger than 18 or older than 65 years, who declined to participate in the study, or who had physical or mental impairments that hindered them from answering the questions. The sampling process is described as follows. In dental school, we selected 30 patients from each desired department (8 departments) using a simple random sampling method. For this purpose, we randomly selected 10 days out of 20 working days in one month and randomly selected three patients from the patient waiting list of each department on each day. For private clinics, we first obtained the names of private clinics in Shiraz city from the Vice-Chancellor of Medical Sciences of Shiraz University of Medical Sciences and randomly selected one or two clinics from each municipal district (11 districts in total), resulting in 20 clinics. Then, we randomly selected two working days for each clinic according to their schedule and selected 10 patients who met the inclusion criteria from each clinic on each day based on their appointment list. After obtaining informed consent from the patients, we asked them to complete a questionnaire in two stages including before and after receiving the treatment service. The first stage aimed to measure the patients' expectations of the treatment service and the second stage aimed to measure their perceptions of SERVQUAL. In this way, we could better distinguish between the expectations before treatment and the perceptions after treatment. We did not evaluate the quality of occupational therapy in this study but rather assessed the entire treatment process from admission to completion of treatment. We used a trained interviewer to administer the questionnaire and provide explanations for illiterate people or clarify any doubts of the patients without changing the essence of the questions. 

The questionnaire used in this study was the SERVQUAL performance gap questionnaire, which was developed and validated in Persian [ 1 , 8 ] for measuring SERVQUAL and satisfaction in healthcare settings. The questionnaire had two parts: demographic information and SERVQUAL dimensions. The demographic information included gender, age, occupation, education level, marital status, and insurance status. SERVQUAL dimensions included six aspects: tangibility, reliability, responsiveness, assurance, empathy, and access. Each dimension had different number of questions as tangibility (5 questions), reliability (7 questions), responsiveness (5 questions), assurance (7 questions), empathy (5 questions), and access (2 questions). The questions were scored on a five-point Likert scale (1= very low, 2= low, 3= medium, 4= high, 5= very high) and the total score of each questionnaire was calculated by summing up all the scores. 

The data collected from the questionnaire were analyzed using descriptive and inferential statistics. Descriptive statistics included the calculation of mean, standard deviation, frequency, and percentage for each item and dimension of the SERVQUAL model. The paired t-test was used to evaluate the gap for each dimension of SERVQUAL. The independent t-test was used to examine the effects of gender, current status, insurance, and the frequency of referral on the gap. The one-way analysis of variance was used to test the effects of education and occupation on the gap. The Tukey post hoc test was used to compare the groups pairwise. The level of significance for all tests was set at 0.05. We entered the collected data into SPSS software version 25 and used Microsoft Excel version 2019 to draw various tables and graphs. 

## Results

As shown in Table 1, the study sampled 440 patients who had a mean age of 35.91±11.04 years. Most participants constituted women 58.2%, were married (73.4%), had a diploma and post-diploma degrees (34.1%), were unemployed (42.5%), and had public insurance (76.8%). The participation in dental school and private clinics was nearly close together (54.5% and 45.5%, respectively). The demographic results of the dental school and private clinic can be seen in Table 1. 

**Table 1:** Demographic information

		Dental School (n=240) 54.5%		Private Clinics (n=200) 45.5%		Total (n=440)	
		N	%	N	%	N	%
Gender	Woman	134	55.8	122	61.0	256	58.2
	Man	106	44.2	78	39.0	184	41.8
Education	High school	58	24.2	18	9.0	76	17.3
	Diploma &PD*	94	39.2	56	28.0	150	34.1
	Bachelor	57	23.8	89	44.5	146	33.2
	Master & Higher	31	12.9	37	18.5	68	15.5
Martial	Single	51	21.3	66	33.0	117	26.6
	Married	189	78.8	134	67.0	323	73.4
Employment	self-employment	91	37.9	66	33.0	157	35.7
	Governmental	43	17.9	53	26.5	96	21.8
	Unemployed	106	44.2	81	40.5	187	42.5
Insurance	Public	199	82.9	139	69.5	338	76.8
	Private	41	17.1	39	19.5	80	18.2
	Non - insurance	199	82.9	22	11.0	22	5

* Diploma and postgraduate diploma

**Figure 1:**
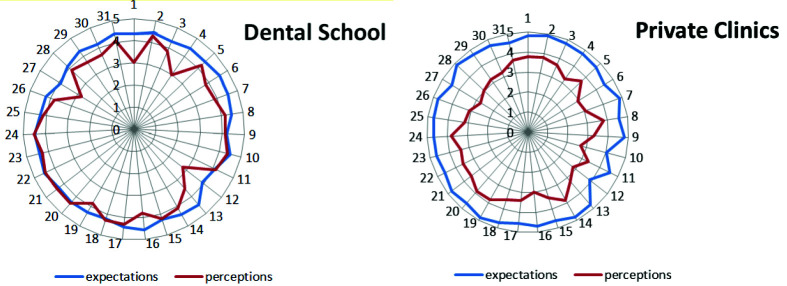
Radar diagram of the average expectations (blue lines) and perceptions (red lines) of patients referring to the dental school and private clinics according to questions of the service quality) SERVQUAL questionnaire. The numbers in the circle of the graph represent questions 1-31 of the SERVQUAL questionnaire and correspond to the numbers of the questions in Table 3 and the numbers in the center according to the average score given by patients are to expectations and perceptions. The gap between dental school (mean =-.40 ±.45) and private clinics (mean =-1.27±.59). The difference gap between the two groups was 0.86±0.51 which was significant (*p*= 0.000)

Table 2 shows the distribution of the dimensions of expectations and perception as well as the gap between them in the studied groups. Total satisfaction (dental school and private clinics) is 84.2%. The average gaps between the dimensions can be seen in Table 2. According to Table 3, all the dimensions had a significant negative gap (*p*= 0.000), but this result was not obtained when examining the questions of the dimensions separately. According to Figure 1 and Table 2, the mean gap between private clinics was 0.86±0.51 more than the dental school which was significant (*p*= 0.000). 

**Table 2:** Mean and standard deviation (SD) of expectation, perception, and gap in the service quality (SERVQUAL) provided

		Dental School		Private Clinics		Total	
		Mean	SD*	Mean	SD	Mean	SD
Tangibility	Expectation	4.40	.43	4.79	.25	4.58	.41
	Perception	3.68	.54	3.62	.78	3.65	.66
	Gap	-.40	.45	-1.27	.59	-0.92	.74
	*p* Value	0.000		0.000		0.000	
Reliability	Expectation	4.32	.39	4.45	.29	4.38	.35
	Perception	3.89	.58	3.14	.59	3.55	.69
	Gap	-.40	.98	-1.15	.95	-0.82	.77
	*p* Value	0.000		0.000		0.000	
Responsiveness	Expectation	4.43	.41	4.68	.31	4.54	.39
	Perception	3.99	.61	3.38	.67	3.71	.71
	Gap	-.50	.71	-1.64	.65	-0.83	.81
	*p* Value	0.000		0.000		0.000	
Assurance	Expectation	4.36	.41	4.70	.23	4.52	.38
	Perception	4.30	.56	3.65	.61	4.01	.66
	Gap	-.05	.65	-1.04	.64	-0.50	.81
	*p* Value	0.160		0.000		0.000	
Empathy	Expectation	4.16	.49	4.69	.29	4.40	.49
	Perception	3.66	.63	3.05	.64	3.38	.70
	Gap	-.44	.64	-1.30	.75	-1.0	.89
	*p* Value	0.000		0.000		0.000	
Access	Expectation	4.29	.63	3.46	.92	3.91	.87
	Perception	3.88	.92	4.61	.32	4.21	.80
	Gap	-.42	.64	-1.31	.61	0.30	1.24
	*p* Value	0.000		0.000		0.000	
Total	Expectation	4.33	.31	4.65	.17	4.48	.30
	Perception	3.93	.48	3.38	.59	3.68	.60
	Gap	-.40	.45	-1.27	.59	-0.79	.68
	*p* Value	0.000		0.000		0.000	

Statistical Analysis with Pair T-test, *SD= Standard Deviation

**Table 3:** The average expectations, perceptions, and the total gap according to the questions of the service quality (SERVQUAL) questionnaire

		Dental School							Private Clinics							Total						
		Exp		Per		Gap			Exp		Per		Gap			Exp		Per		Gap		
D	Question	M	SD	M	SD	M	SD	P	M	SD	M	SD	M	SD	P	M	SD	M	SD	M	SD	P
Tangibility	Attractive physical environment and guidance signs	4.32	.66	3.03	.81	-1.29	1.07	.000	4.81	.39	3.76	.90	-1.05	.96	.000	4.54	.60	3.36	.93	-1.18	1.03	.000
	Doctors and staff with a clean and orderly appearance	4.48	.57	4.30	.77	-.18	.87	.001	4.90	.30	3.81	1.01	-1.09	1.10	.000	4.67	.51	4.07	.92	-.59	1.08	.000
	Appropriate and up-to-date medical equipment	4.33	.86	3.85	.92	-.47	1.19	.000	4.80	.39	3.64	.92	-1.16	.93	.000	4.54	.73	3.75	.92	-.78	1.14	.000
	Presence of visible and attractive guidance signs	4.46	.66	3.00	.82	-1.45	1.08	.000	4.76	.42	3.22	.98	-1.54	1.09	.000	4.60	.58	3.10	.90	-1.49	1.08	.000
	Comfortable and clean waiting room and bed	4.43	.58	4.21	.81	-.22	.89	.000	4.69	.46	3.68	.86	-1.01	.94	.000	4.55	.54	3.97	.87	-.58	.99	.000
Reliability	Providing services on time	4.57	.59	3.81	1.12	-.75	1.29	.000	4.47	.50	2.95	.80	-1.52	.92	.000	4.52	.55	3.42	1.08	-1.10	1.20	.000
	Providing services at the right time and on time	4.52	.61	3.90	1.04	-.62	1.23	.000	4.87	.33	3.06	.80	-1.81	.90	.000	4.68	.53	3.52	1.03	-1.16	1.24	.000
	The professionalism of doctors and staff	4.45	.64	4.16	.81	-.29	.98	.000	4.57	.49	3.82	.93	-.74	1.07	.000	4.50	.58	4.01	.88	-.49	1.05	.000
	Provide documents without mistakes and as soon as possible	4.21	.76	4.13	.81	-.07	1.02	.231	4.82	.38	3.30	.96	-1.52	.961	.000	4.49	.69	3.75	.97	-.73	1.22	.000
	Logical relationship between cost and service	4.49	.59	4.35	.81	-.13	.87	.016	4.04	.79	2.70	.85	-1.34	1.08	.000	4.28	.72	3.60	1.17	-.68	1.14	.000
	Detailed information about the service delivery process	4.07	.78	4.12	.90	.04	1.08	.513	4.53	.52	3.34	.89	-1.19	.96	.000	4.28	.71	3.76	.98	-.51	1.20	.000
	Access to nurse and dentist at night	3.92	.84	2.79	1.04	-1.13	1.27	.000	3.87	.78	2.82	1.35	-1.05	1.54	.000	3.90	.81	2.80	1.19	-1.09	1.39	.000
Responsiveness	Provide fast service to patients	4.49	.60	3.53	1.22	-.95	1.26	.000	4.76	.42	3.24	.85	-1.52	.98	.000	4.61	.54	3.40	1.08	-1.21	1.17	.000
	The encounter of dentists in building trust	4.41	.64	4.09	.81	-.32	.97	.000	4.81	.38	3.86	.84	-.95	.94	.000	4.59	.58	3.98	.83	-.60	1.01	.000
	Willingness to be helped by clinic staff	4.27	.63	4.23	.74	-.04	.87	.460	4.59	.49	3.40	.84	-1.19	.97	.000	4.42	.59	3.85	.89	-.56	1.08	.000
	Waiting time of less than an hour	4.55	.62	3.81	1.20	-.73	1.28	.000	4.71	.45	2.99	.86	-1.72	1.03	.000	4.62	.55	3.44	1.14	-1.18	1.27	.000
	Necessary guidance by the receptionist	4.45	.67	4.30	.88	-.15	.99	.021	4.54	.49	3.41	1.04	-1.13	1.19	.000	4.49	.60	3.90	1.06	-.59	1.19	.000
Assurance	Humble attitude of staff and dentists	4.23	.66	4.29	.80	.05	1.02	.381	4.70	.45	3.52	.86	-1.17	.93	.000	4.44	.62	3.94	.91	-.50	1.16	.000
	Dentist's knowledge in treating patients	4.29	.69	3.83	.89	-.46	1.14	.000	4.87	.33	3.83	.90	-1.03	.95	.000	4.55	.62	3.83	.89	-.72	1.09	.000
	Observance of human relations with patients	4.34	.68	4.41	.69	.07	.90	.227	4.65	.47	3.86	.80	-.79	.93	.000	4.48	.61	4.16	.79	-.32	1.01	.000
	Full description of medical conditions and illnesses	4.29	.74	4.37	.75	.08	1.00	.177	4.78	.41	3.54	.71	-1.24	.83	.000	4.51	.66	3.99	.84	-.51	1.13	.000
	Respect patient privacy	4.50	.65	4.47	.67	-.02	.90	.617	4.59	.49	3.58	.82	-1.00	.94	.000	4.54	.59	4.07	.86	-.47	1.04	.000
	Answering patients' questions	4.41	.64	4.25	.78	-.15	.89	.006	4.67	.46	3.44	.74	-1.23	.88	.000	4.53	.58	3.88	.86	-.64	1.03	.000
	Feeling safe in the clinic	4.48	.61	4.50	.64	.01	.77	.740	4.68	.46	3.82	.92	-.85	1.00	.000	4.57	.55	4.19	.85	-.37	.98	.000
Empathy	Listening to patients' ideas and opinions	4.27	.73	4.14	.87	-.12	1.02	.052	4.73	.44	3.18	.74	-1.55	.75	.000	4.47	.66	3.70	.94	-.77	1.15	.000
	Get feedback from patients	4.25	.70	3.83	.83	-.42	.96	.000	4.74	.43	3.09	.86	-1.65	1.00	.000	4.47	.64	3.49	.92	-.98	1.15	.000
	24/7 service	3.91	.83	2.79	1.03	-1.1	1.27	.000	4.43	.69	2.75	1.14	-1.68	1.29	.000	4.15	.81	2.77	1.08	-1.37	1.31	.000
	Showing interest in patients	4.12	.75	3.88	.88	-.24	.97	.000	4.85	.35	3.02	.83	-1.82	.87	.000	4.45	.70	3.49	.96	-.96	1.21	.000
	Attention to the specific needs of patients	4.28	.70	3.69	.91	-.59	1.05	.000	4.71	.45	3.21	.79	-1.50	.87	.000	4.47	.63	3.47	.89	-1.00	1.07	.000
Access	Suitable facilities for patients' companions	4.17	.77	3.67	1.03	-.49	1.15	.000	4.69	.46	3.24	1.13	-1.45	1.15	.000	4.40	.70	3.47	1.10	-.92	1.25	.000
	Availability of the place of payment of fees	4.41	.70	4.09	.99	-.31	1.05	.000	4.54	.49	3.68	1.06	-.86	1.19	.000	4.47	.62	3.90	1.04	-.56	1.15	.000

Statistical Analysis with Pair T-test, D: Dimension, Exp: Expectation, M: Mean, P: *p* Value, Per: Perception, SD: Standard Deviation

### Dental school 

The study examined the satisfaction of patients (92%) with SERVQUAL at dental school. The results indicated that the patients had high expectations and low perceptions of SERVQUAL, leading to significant gaps in all dimensions (*p*= 0.000). The highest expectation score was for responsiveness (4.43±0.41), while the lowest was for empathy (4.16±49). The highest perception score was for assurance (4.30±0.56), while the lowest was for empathy (3.66±0.63). The highest gap score was for responsiveness (-0.50±0.71), while the lowest was for reliability (-0.05±0.65). The questions related to providing services on time (4.57±0.59) and” feeling safe in the clinic” (4.50±0.64) had the highest mean scores of expectations and perceptions, respectively, while the questions related to “24/7 service” (3.91±0.83)” access to nurse and dentist at night” (2.79±1.03) with “24/7 service” (2.79±1.04) both jointly had the lowest mean scores of expectations and perceptions, respectively. The question related to “full description of medical conditions and illnesses “(0.8±1.00) had the highest mean score of the gap, while the question related to “presence of visible and attractive guidance signs “(-1.45±1.08) had the lowest mean score of the gap (Table 3). All questions had a significant gap (*p*= 0.000) except three questions (willingness to be helped by clinic staff, detailed information about the service delivery process, providing documents without mistakes and as soon as possible).

The statistical analysis showed that the average score in expectations and gap of married people in dental school was higher than that of single people in all dimensions (*p*< 0.05). In addition, the expectations regarding access to government jobs are significantly higher than the expectations of those with freelance jobs (*p*< 0.05). There were two significant and direct relationships between age and patients' expectations (empathy r= 0.164, *p*= 0.011 and access r= 0.287, *p*< 0.001) and an inverse relationship in the reliability dimension (r=0.178 and *p*=0.006). There were two significant and reverse relationships between age and patients' perception (empathy r= -0.183, *p*= 0.004 and access r= -0.160, *p*= 0.013). There were two significant and reverse relationships between age and patients' gap (empathy r=-0.275, *p*= 0.000 and access r=-0.333, *p*= 0.000).

### Private clinics 

The results of the study indicated that the patients in private clinics had high expectations and low perceptions of SERVQUAL, resulting in a significant negative gap in all dimensions. The dimensions of tangibility (4.79±0.25) and access (4.61±0.32) had the highest mean scores of expectations and perceptions, respectively, while the dimensions of empathy (3.05±0.64) and access (3.46±0.92) had the lowest mean scores of expectations and perceptions, respectively. The questions related to the appearance of the doctors and staff (4.90±0.30), the encounter of the dentists in building trust (3.86±0.84), and the observance of human relations with patients (3.86±0.80) had the highest mean scores of expectations and perceptions, respectively, while the questions related to the access to nurse and dentist at night (3.87±0.78), the logical relationship between cost and service (2.70±0.85) had the lowest mean scores of expectations and perceptions, respectively. The dimension of responsiveness (-1.64±0.65) had the highest mean score of the gap, while the dimension of reliability (-1.15±0.95) had the lowest mean score of the gap. The question related to showing interest in patients (-1.82±0.87) had the highest mean score of the gap, while the question related to the professionalism of doctors and staff (-0.74±1.07) had the lowest mean score of the gap (Table 3). The results of the study also showed that 74.6% of the patients in private clinics were satisfied with the service. The results of the statistical analysis showed that the gap in the dimension of assurance was greater in unemployed people than in people with freelance jobs (p&lt; 0.05). In addition, a significant difference was found between the expectations of education levels in the dimension of assurance (*p*= 0.004), and between the perceptions and gaps of education levels in the dimension of responsiveness (p&lt; 0.05).

Finally, except for the mentioned statistical results, did not find any significant relation in both settings (*p*> 0.05). 

## Discussion

The purpose of this study is to investigate and compare the quality of dental services provided in Shiraz dental school and private clinics based on patients' feedback. These results show how patients rate the quality of services they receive and how their ratings are related to their characteristics. Based on the SERVQUAL model, the results compare the ability of patients to express their expectations (what they hoped to receive) and perceptions (what they received) of the service, and then the gap (difference) is calculated. A negative gap means that perception was lower than expected, indicating dissatisfaction. A positive gap means that perception was higher than expected, which indicates satisfaction. A gap of zero means that perception matches expectation, indicating neutrality [ 13 ]. Considering the importance of patient satisfaction and identifying their expectations and perceptions of SERVQUAL, this study has investigated the role of various factors on the level of satisfaction of patients referring to dental faculty and private clinics in Shiraz. The results of this study showed that the patients were generally satisfied with the services provided, but there is a negative gap and the patient's expectations have not been met significantly in all dimensions, and the services provided are less than the patients' expectations in Shiraz. Previous studies mentioned in this study mostly have focused on educational centers in Iran [ 1 , 3 - 4 , 7 , 14 - 15 ]. Haji Fatahi *et al*. [ 3 ] reported a 71% satisfaction level among patients referred to the dental clinics of the Islamic Azad University of Tehran. Razmi and Jafari [ 16 ] reported a satisfaction level of 57.8% among patients referred to the Faculty of Dentistry of Tehran University of Medical Sciences. Crossley *et al*. [ 17 ] reported a satisfaction level of 89% among patients referred to the University of Manchester Dental Centre, England. Ghapanchi *et al*. [ 7 ] reported a satisfaction level of 87.5% among patients referred to Shiraz Dental School in 2008. It seems that the difference in the satisfaction index in this research may be due to the investigated factors, the method of data collection, the use of different questionnaires, and the variety of questions and rating scales [ 18 ]. According to this study, there is a negative gap between patients' expectations and perceptions in all dimensions except access. This finding shows that most dimensions do not meet the expectations of patients. Teshnizi *et al*. [ 19 ] reported in a meta-analytical study of quality assessment of healthcare services with the SERVQUAL model in Iran that Shiraz and another city in Iran had the largest gap between patients' expectations. Bastani *et al*. [ 8 ] reported a significant gap in all these dimensions in the satisfaction survey of patients referred to Martyr Ayatollah Motahari Clinic in Shiraz in 2013. The results show that patients had high expectations and low understanding of SERVQUAL in all dimensions, which resulted in significant negative gaps. This means that the patients were not satisfied with the services received and did not meet their expectations. The results also show that patients valued different aspects of the service differently, and some aspects had larger gaps than the rest. It appears that the provision of services in the city of Shiraz in the past years and this study still has a significant gap [ 7-8, 19]. 

Dental school patients had the highest expectations for responsiveness, meaning they wanted providers to be prompt, attentive, and helpful. However, they had the lowest perception of empathy, meaning they felt service providers did not care about their needs, feelings, and preferences. In a similar study that was conducted in Tehran, the empathy dimension also had the largest gap, which may be due to the lack of initial treatment and sufficient respect for patients despite the efforts of dentists in educating and asking patients [ 4 ]. This dimension is very important for creating empathy and getting the patient's cooperation in accepting and continuing the treatment, so it needs special attention [ 1 ]. The largest gap was also related to responsiveness, meaning that patients were most dissatisfied with this aspect of the service. The smallest gap was related to reliability, meaning that patients were least satisfied with this aspect of the service. In addition, the results show that some specific questions had higher or lower scores than others. Patients had the highest expectations from the question "providing services on time", which could be related to the history of receiving services or long queues for providing services in government systems. The most satisfaction was related to the feeling of security that could be due to creating a calm environment and monitoring the behavior of employees [ 1 , 3 - 4 , 7 ]. On the other hand, patients were the least satisfied with the questions "access to nurse and dentist at night" and "24-hour service", which had the lowest scores in terms of both expectation and perception, possibly due to patients' awareness of the limited hours during which government services are provided [ 20 ]. The question that had the biggest gap was "full explanation of conditions and diseases", meaning that patients expected more information and explanations from service providers. The question that had the least distance was "the existence of visible and attractive guidance signs", which means that the patients did not expect much from this aspect of the service. 

Private clinic patients had the highest expectations for tangibility, meaning they wanted service providers to have good appearance, equipment, and facilities. However, they had the lowest perception of empathy, meaning they felt service providers did not care about their needs, feelings, and preferences. In the dimension of access, the least expectation and the most understanding were included, which seems that private clinics have facilitated the conditions for access of patients [ 21 ]. The largest gap was also related to responsiveness, meaning that patients were most dissatisfied with this aspect of the service. The smallest gap was related to reliability, meaning that patients were least satisfied with this aspect of the service. Moreover, the results show that some specific questions had higher or lower scores than others. The patients had the most expectations from the question "appearance of doctors and staff". In addition, the most satisfaction was related to the treatment based on trust and respect for human relations, which a good relationship between doctors and patients depends on the patient's confidence in the doctors, the quality of the communication, and the mutual respect that was well done in private clinics [ 22 ]. On the other hand, patients had the least expectations from the questions "access to nurse and dentist at night" and the least satisfaction from "rational relationship between cost and service". The question with the largest gap was "showing interest in patients", meaning that patients expected more attention and involvement from service providers [ 23 ]. The question that had the least gap was "professionalism of doctors and personnel", which means that patients did not have much expectation or perception for this aspect of the service and that they were neutral or slightly satisfied with it [ 24 ]. 

Statistical analysis of demographic variables shows that patients' rating of SERVQUAL was influenced by their demographic and social factors such as marital status, occupation, education level, and age. The results show that married patients were less demanding and satisfied than single patients, and patients who had government jobs wanted more convenience and availability from service providers than those who had freelance jobs. Additionally, the results show that age has a significant and complex relationship with patients' expectations, perceptions, and gaps. Older patients had higher expectations for empathy and access compared to younger patients; however, they had lower perceptions of empathy and access, and wanted more personal attention and easier access from providers, but felt they were not getting those [ 25 ].Furthermore, younger patients wanted more consistency and speed from service providers but felt they were not getting those [ 26 ]. Danesh Kazemi *et al*. [ 5 ] found that the level of satisfaction decreases with age and attributed this to a decrease in patience in the elderly. They were more dissatisfied with the competence, politeness, and security of the service providers. In addition, the level of education has had a significant effect on the expectations, perceptions, and gaps of patients in terms of confidence and patient responsiveness. Patients with higher levels of education had higher expectations for reassurance, meaning they wanted more trust and credibility from service providers than the patients with lower levels of education. In the study of Puriene *et al*. [ 27 ], patients preferred the private dental sector due to the better quality of services, the public - due to the proximity to the place of residence, or the treatment of acquaintances. Patients visiting public institutions often seek more affordable treatments, whereas those visiting private institutions tend to use modern, high-quality, albeit more expensive, technologies. Last year, the number of dental visits in public institutions was lower than in private institutions [ 1 , 4 ]. Older patients visit general dental institutions more frequently than younger individuals. More patients are treated in public dental institutions than in private ones. Older patients, who typically have lower incomes, prefer public institutions [ 2 - 6 ]. 

Rokhshani *et al*. [ 28 ] also measured patient satisfaction and the quality of healthcare services based on the SERVQUAL model in Ahvaz healthcare centers in Iran. Their findings showed that patient satisfaction and the quality of dental health services were influenced by age, gender, type of treatment, cost, and time of treatment. The highest score of expected and perceived SERVQUAL was related to tangibility and accessibility, and the lowest score was related to empathy and accessibility. The highest gap score was related to responsiveness and the lowest was related to reliability. The highest level of satisfaction was "dentists' encounter in building trust" and the lowest level of satisfaction was "access to nurses and dentists at night". These findings had similarities with the present study, such as the existence of a gap between expectations and perceptions, the effect of demographic and social factors on SERVQUAL and patient satisfaction, and the diversity of SERVQUAL and patient satisfaction in service delivery. 

The SERVQUAL model is a widely used and useful tool for evaluating service quality and patient satisfaction in dental healthcare environments. SERVQUAL is an important factor for patient loyalty and retention [ 1 ]. The quality of service and patient satisfaction varies depending on the context, sample, and dimensions of the model. It is influenced by the personal characteristics of patients, such as educational background, occupational groups, health insurance, income level, and the reason for choosing the clinic [ 2 ]. The quality of service and patient satisfaction has common and specific areas for improvement, such as responsiveness, empathy, cost, and access [ 3 ]. We suggested different settings and developed strategies to enhance the perceived value of services in future studies [ 1 , 4 ]. 

Finally, SERVQUAL and patient satisfaction in dental health environments are complex and dynamic phenomena that require regular and systematic measurement and evaluation, and service providers must consider patients' needs and preferences in designing and providing services [ 29 - 31 ]. The limitations of this study included the low willingness of the patients to participate in completing the questionnaires, the time-consuming nature of recording information, and the low literacy level of some patients and the difficulty of understanding some of the criteria of the questionnaire, the unavailability of many patients after the procedure. Additionally, the treatment does not take into account the opinions of students, faculty members, doctors, and medical personnel when assessing the quality level. 

## Conclusion

The quality of dental services provided in Shiraz Dental School and private clinics is satisfactory, but there is room for improvement in both settings. The service providers should pay more attention to the expectations and perceptions of the patients, and to the factors that affect their satisfaction. The study provides useful information for the managers and policymakers of the dental sector, as well as for the students and practitioners of dentistry. The study also contributes to the literature on SERVQUAL and satisfaction in healthcare settings, especially in the context of Iran. 
